# Who stop telemonitoring disease activity and who adhere: a prospective cohort study of patients with inflammatory arthritis

**DOI:** 10.1186/s41927-022-00303-w

**Published:** 2022-11-30

**Authors:** J. Wiegel, B. F. Seppen, M. T. Nurmohamed, W. H. Bos, M. M. ter Wee

**Affiliations:** 1 Amsterdam Rheumatology and Immunology Center, Reade, Amsterdam, Admiraal Helfrichstraat 1, 1056 AA Amsterdam, The Netherlands; 2 Rheumatology and Immunology, Amsterdam UMC Location Vrije Universiteit Amsterdam, Amsterdam, The Netherlands; 3 Epidemiology and Data Science, Amsterdam UMC Location Vrije Universiteit Amsterdam, Amsterdam, The Netherlands; 4 Societal Participation and Health, Amsterdam Public Health, Amsterdam, The Netherlands; 5 Amsterdam Public Health, Methodology, Amsterdam, The Netherlands; 6 Amsterdam Infection and Immunity, Amsterdam, The Netherlands

**Keywords:** Adherence, Telemonitoring, Telehealth, ePRO’s, Inflammatory arthritis, Patient reported outcomes, Dropout

## Abstract

**Background:**

The use of frequent electronic patient reported outcome measures (ePRO’s) enables monitoring disease activity at a distance (telemonitoring) in patients with inflammatory arthritis. However, telemonitoring studies report declining long-term adherence to reporting ePRO’s, which may oppose the benefits of telemonitoring. Therefore, the objective was to investigate what factors are associated with (non-)adherence to telemonitoring with a weekly ePRO in patients with inflammatory arthritis (IA).

**Methods:**

We performed a prospective cohort study in patients with rheumatoid arthritis (RA), psoriatic arthritis (PsA) and ankylosing spondylitis (AS) at Reade Amsterdam, The Netherlands. Patients telemonitored their disease activity weekly for 6 months with a modified Multidimensional Health Assessment Questionnaire completed in a smartphone application. The primary outcome was time to dropout, defined as ≥ 4 weeks of consecutively nonresponse. Based on literature and through expert meetings, a predefined set of 13 baseline factors were selected to assess the association with time to dropout through a multivariable Cox-regression analysis.

**Results:**

A total of 220 consecutive patients were included (mean age 54, SD 12; 55% females; 99 RA, 81 PsA, and 40 AS). A total of 141 patients (64%) dropped out, with a median time to dropout of 17 weeks (IQR 9–26). Women had a significant higher chance to dropout over 6 months compared to men (HR 1.58, 95% CI 1.06–2.36).

**Conclusion:**

In the set of investigated factors, women stopped reporting the weekly ePRO sooner than men. Future focus group discussions will be performed to investigate the reasons for dropout, and in specific why women dropped out sooner.

*Trial registration* This trials was prospectively registered at www.trialregister.nl (NL8414).

**Supplementary Information:**

The online version contains supplementary material available at 10.1186/s41927-022-00303-w.

## Background

Inflammatory arthritis demands long-term disease activity monitoring [1] which requires an outpatient clinic visit to assess the disease activity with composite measures such as the Disease Activity Score 28 (DAS28) or Clinical Disease Activity Index (CDAI) [2, 3]. However, with electronic patient reported outcomes (ePRO’s) the disease activity can be measured longitudinally at a distance (telemonitoring) in between visits. This leads to a reduction of clinical visits while maintaining tight disease control, and higher patient satisfaction regarding both shared decision making and physician’s awareness of disease fluctuations [4–6].

Although high adherence to reporting ePRO’s through mobile applications (apps) was observed in recently performed trials that investigated telemonitoring, often adherence declined over time reducing the potential benefits [7–9]. For example, adherence decreased from 88 to 62% during the 6 month study of Lee et al. [10]. In addition, Seppen et al. reported declining adherence rates from > 90% in week one, to less than 50% in week four [11]. Identifying factors related to nonadherence increases our understanding of adherence to reporting ePRO’s over time and may identify factors that can be influenced to improve adherence.

Multiple models such as the Unified Theory of Acceptance and Use of Technology (UTAUT) and Technology Acceptance Model (TAM) are constructed to explain adoption and usage behavior of new technologies [12, 13]. A recent systematic review regarding adherence of telemonitoring with ePRO’s in patients with chronic diseases showed that lower eHealth literacy (the ability to seek and use health information from electronic resources) and the presence of comorbidity may act as potential barriers to adherence [14]. Within rheumatology specifically, Colls et al. retrospectively found in rheumatoid arthritis (RA) patients that a higher disease activity was associated with lower adherence to reporting ePRO’s, and being older than 65 years with higher adherence [15]. Other presumed important factors such as gender and educational level were not significantly associated to adherence. During a qualitative study, receiving appropriate training, clarity of instructions and a simple user interface of a mobile application (app), were identified as potential facilitators for higher adherence. Experiencing technical issues with the app were potential barriers for adherence [9, 16]. To conclude, evidence regarding which factors influence adherence to reporting ePRO’s is limited, and often retrospectively tested, univariably or in a limited combined set of factors. But, comparable with medication compliance, adherence is a complex concept in which a multitude of factors correlate with adherence and possibly each other.

Therefore, the objective of this study was to prospectively explore the association between a combined set of patients- and clinical related factors with (non)-adherence to telemonitoring with a weekly ePRO through a smartphone app, in patients with inflammatory arthritis.

## Methods

### Study design

We performed a 6-month prospective cohort study at Reade, a center for rehabilitation and rheumatology, in Amsterdam, The Netherlands, from April 2020 to June 2021. The protocol was registered at ICTRP Search Portal (who.int) (NL8414) at 28-02-2020. Patients continued routine clinical care, with the addition of weekly telemonitoring of their disease activity through an ePRO questionnaire that was completed in a smartphone application designed specifically for this purpose: the MijnReuma Reade app [11].

The app has already been extensively described in previous publications [11, 17]. In short, the MijnReuma Reade app was developed at Reade with the aim to enable patients to monitor their symptoms and disease activity on a weekly base at home with a modified version of the Multi-Dimensional Healthcare Assessment Questionnaire (MDHAQ) with the addition of a single flare question, see Table 1 [17, 18]. The results were displayed in text supported by graphs for disease activity, pain, function, overall wellbeing, fatigue, and morning stiffness. Patients received a badge notification when the new ePRO questionnaire was available, and a reminder was sent after three days when the ePRO questionnaire was not yet filled in. The app transferred the patients' data in real-time to their Electronic Medical Record (EMR) at Reade, making the results directly visible for the health care providers at Reade. The app was secured by a two factor authentication, is CE certified, and compliant with the Dutch privacy and security laws [19].

**Table 1:** The weekly ePRO questionnaires included in the MijnReuma Reade app (17)

Domain	Measure	Questions
Function^a,b^	Likert scale (0–3)	10
Pain^a,b^	NRS (0–10)	1
Patient-global^a,b^	NRS (0–10)	1
Fatigue^b^	NRS (0–10)	1
Morning stiffness^b^	Minutes	1
Social participation^b^	Likert scale (0–3)	1
Sleep^b^	Likert scale (0–3)	1
Anxiety^b^	Likert scale (0–3)	1
Stress^b^	Likert scale (0–3)	1
Flare question^c^	Yes/no	1

*NRS* Numeric Rating Scale

^a^Function, pain and patient-global composes the Routine Assessement of Patient Index Data 3 (RAPID3)

^b^Part of the MDHAQ

^c^Single question: “Do you experience currently a flare in disease activity?”

### Study outcome

Adherence and nonadherence to reporting ePRO’s was measured after 6 months. According to the adherence framework of O’Brien et al., we considered multiple metrics to define (non-)adherence [20]. We deemed time to dropout as the most suitable metric for non-adherence as the primary outcome for this study. Dropout was defined as ≥ 4 weeks of consecutively nonresponse to the ePRO, based on the 0.9 percentile of all average user gaps [21]. Adherence was the secondary outcome, measured as the User Activity Ratio (UAR). The UAR is the number of reported ePRO’s divided by the number of potentially reported ePRO’s (26 in this study) × 100.

### Patient selection and recruitment procedure

To minimize selection bias, and to increase the generalizability of our future findings, we consecutively approached all patients with Rheumatoid Arthritis (RA), Psoriatic Arthritis (PsA) or Ankylosing Spondylitis (AS) who had a physical or telephone consultation with their rheumatologist at the outpatient clinic, from March 2020 until December 2020. The inclusion criteria were: (1) diagnosed with RA, PsA or AS according to their rheumatologist, (2) having an Android or iOS-based smartphone, and (3) able to speak, read and write Dutch. The exclusion criteria were (1) not having an e-mail address and (2) previously participating in a trial in which the “MijnReuma Reade” app was used. Due to local COVID-19 regulations, the study was performed without any face-to-face contact between patients and the researchers. Patients were invited by e-mail including detailed information about the study. One week thereafter, the researcher phoned the patients to ask if they were interested in participation and to answer any questions regarding the study. Additional information (phone number and email address) was given about how to contact the researchers when participants encountered any (technical) problems with the app. If the patient consented, additional instructions were given on how to use the app. Personal log-in credentials along with a link to the iOS store and Google Play store to download the app, were sent to the patient per e-mail. When the patient logged-in for the first time, they were asked to fill in the electronic informed consent file in the app through a checkbox. Patients were only definitively included in the study when both the oral and electronic informed consent were obtained. This study is performed in accordance with relevant guidelines and regulations, and in specific the legislation of the medical ethics committee of the Vrije Universiteit medisch centum (VUmc) at Amsterdam, the Netherlands (case number 2019.641), who issued a waiver for this study at 05-11-2019. All methods were carried out in accordance with relevant guidelines and regulations (declaration of helsinki).

### Investigated factors

To select a predefined set of factors that we were interested in to see if these factors were associated with (non)-adherence, we could not rely solely on existing comparable studies which investigated adherence to telemonitoring by ePRO’s in the field of rheumatology, as these are scarce. Therefore, we examined established models describing how users come to accept and use a technology, such as the TAM and UTAUT, and selected candidate factors based on these models [12, 13]. We complemented the list with possible relevant clinical and sociodemographic factors identified through consensus meetings between JW, BS, and WB. The final set of investigated factors is shown in Table 2.

**Table 2:** Set of investigated patient and clinical factors

Factor	Time of measurement	Instrument	Scale
*Patient factors*			
Gender	Baseline		Male/female
Age	Baseline		Years
Education level^a^	Baseline		Low/high
Residing distance to Reade	Baseline		In/out of Amsterdam
Self-management skills	Baseline	EC-17	0–100
Perceived efficacy in patient-physician interaction	Baseline	PEPPI-5	5–25
Smartphone usage	Baseline	ssMTUAS	0–10
Perceived usability of the app	Month 3	SUS	0–100
*Clinical factors*			
Diagnosis	Baseline		RA/PsA/AS
Disease duration	Baseline		Years
Disease activity	Baseline	RAPID3	0–10
Comorbidity	Baseline	CCI	0–20
Medication adherence	Baseline	CQR	Low/high
Biological usage	Baseline		csDMARD only/bDMARD

^a^Completed higher vocational education, university bachelor or higher was defined as high

*EC-17* Effective Consumer Scale 17, *PEPPI-5* Perceived Efficacy in Patient-Physician Interaction 5, *ssMTUAS* smartphone subscale of the Media and Technology Usage and Attitude Scale, *SUS* System Usability Scale, *RAPID3* Routine Assessment of Patient Index Data 3, *CCI* Charlson Comorbidity Index, *CQR* Compliance questionnaire Rheumatology

#### Patient factors

As can be seen in Table 2, a total of nine patient factors were studied. The Effective Consumer Scale 17 (EC-17) measures the skills and behaviors people need to effectively manage their healthcare and consists of 17 items scored on a 5-point Likert scale ranging from 0 (never) to 4 (always)[22]. Higher scores represent more effective patient’s self-management attitude and behavior. The Perceived Efficacy in Patient-Physician Interaction 5 (PEPPI-5) measures the efficacy of patients to interact with their physicians with a 5-item questionnaire [23]. Each item is scored on a 5-point Likert scale ranging from 1 (strongly disagree) to 5 (strongly agree). Higher scores represent higher perceived self-efficacy in patient-physician interaction.

No Dutch eHealth or technology literacy measure exist yet. Therefore we extracted the 10-item smartphone subscale of the Media and Technology Usage and Attitude Scale (MTUAS) and translated it to Dutch (following the guidelines for translation of questionnaires) [24]. The smartphone subscale is validated as an independent questionnaire. Higher scores represent higher smartphone usage. The System Usability Scale (SUS) consists of 10 questions measured on a 5-point Likert scale ranging from 1 (strongly disagree) to 5 (strongly agree) [25]. Higher scores represents a higher perceived usability of the app, a score higher than 68 is considered above average.

#### Clinical factors

A total of six clinical predictors were assessed. Disease activity was measured with the Routine Assessment of Patient Index Data 3 (RAPID3), a composite measure containing the function, pain, and patient global scales derived from the MDHAQ [26]. Comorbidity was measured by the Charlson Comorbidity Index (CCI) [27]. Each comorbidity category has an associated weight, and the sum of all comorbidity weights result in a score. Higher scores predict higher mortality and higher resource usage. A score of zero means that no comorbidities were found.

Medication adherence was measured by the Compliance Questionnaire Rheumatology (CQR) and is a five question self-report medication adherence measure created specifically for patients with rheumatic diseases and discriminates patients in low or high medication adherence groups [28].

### Sample size

We calculated the needed sample size without the estimation of an effect size, since there was no comparative study on which we could estimate the possible effect size. A dropout over time rate of 60% was expected based on the adherence data of a pilot telemonitoring study and preliminary data of an RCT both performed at Reade Amsterdam [5, 11]. Following the method for sample size calculation of Green et al., a minimum of 131 cases were necessary to study 13 factors [29]. We divided the cases (131) by the expected dropout over time (0.60) and concluded that a minimum of 219 participants was needed.

### Statistical analysis

Descriptive values were presented as mean and standard deviation (SD) if normally distributed, otherwise the median and interquartile range (IQR) was presented.

For the primary outcome, the association between the combined set of factors and dropout over time was assessed through a multi-variable Cox proportional hazard regression analysis. In advance, each independent variable was checked for multicollinearity. If multicollinearity was present (variance inflation factor (VIF) > 5), the most relevant factors were chosen based on literature and clinical expertise to remain in the multivariable regression model. Results are presented as hazard ratios (HR) with 95% confidence interval (95% CI). The SUS-score was measured at 3 months and could therefore not be included in the Cox-Regression analysis. We performed a univariable linear regression analysis to compare the SUS-scores for adherent patients, patients dropped out in month 1, month 2–3 and month 4–6.

For the secondary outcome, the association between the combined set of factors and a higher UAR (more reported ePRO’s) was assessed through a multivariable logistic generalized estimating equation (GEE) model. The longitudinal GEE analysis corrects for dependent observations within a person. This was necessary as each patient had 26 observations: for each week the outcome (reported ePRO yes/no) was determined. All factors were selected for the multivariable logistic GEE model because of the large number of records and sufficient number of factors. Results are presented as odds-ratio (OR) with 95% CI for reporting more ePRO’s. All analyses were run on IBM SPSS statistics V23.

### Role of the funding source

This research is investigator-initiated and funded by Pfizer, Sanofi, Eli-Lilly and Novartis. The funders had no role in the design of this study, nor during its execution, analysis, interpretation of the data, or decision to submit results.

## Results

**Fig. 1:**
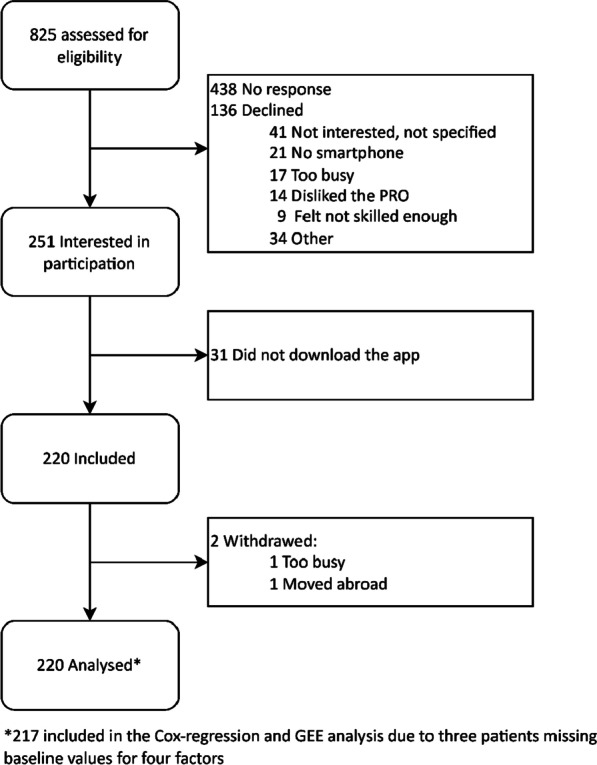
Flow chart. Patients selection and flow through the study

Between April 2020 and December 2020, a total of 825 patients were consecutively assessed for eligibility, of which 220 patients downloaded the app and gave digital informed consent, see Fig. 1. Two patients withdrew during the study: one moved abroad at week seven, and one indicated not having time to continue with the study at week 11. Both patients were included in the analysis, and were considered as dropout as soon as they withdrew. Three patients did not fill out the baseline questionnaire, therefore data regarding their medication adherence, smartphone usage, self-management and patient-physician interaction was missing and were excluded for the Cox-regression and GEE analysis. We included 99 RA patients, 81 PsA and 40 AS patients. The average age was 54 years (SD 12), 55% was female, and the median RAPID3 disease activity was moderate (3.7), see Table 3.

**Table 3:** Baseline characteristics (n = 220)

Female	121 (55%)
Age (years)	54 (13)
Education level, n (%)	
High	140 (64%)
Low	80 (36%)
Resident in Amsterdam, n (%)	99 (45%)
Diagnosis	
Rheumatoid arthritis	99 (45%)
Psoriatic arthritis	81 (37%)
Ankylosing spondylitis	40 (18%)
Charlson Comorbidity Index	2.3 (1,2)
Disease duration in years, median (IQR)	9 (3–19)
Biological usage, n (%)	
b/tsDMARD	111 (50%)
csDMARD only	109 (50%)
RAPID3 baseline, median (IQR)	3.7 (1.6–5.2)
Medication adherence, n (%)*	
High	115 (53%)
Low	102 (46%)
Smartphone usage (1–10)*	4 (2)
Self-management (1–100)*	76 (12)
Patient-physician Interaction (1–25)*	21 (3)

*IQR* Inter-quartile range; *b/tsDMARD* biological/targeted synthetic Disease-Modifying Antirheumatic Drugs; *RAPID3* Routine Assessment of Patient Index Data 3

^*^n = 217. Units are presented as the mean with standard deviation, or otherwise specified

### Time to dropout

**Fig. 2:**
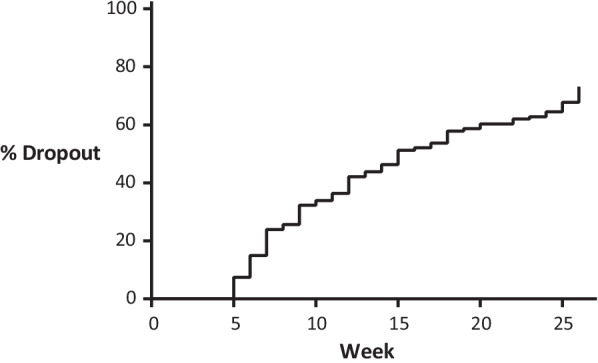
Proportion of participants who stop reporting ePRO’s over time

A total of 79 patients (36%) continued telemonitoring during the 6 months period, and 141 (64%) dropped out (Fig. 2). Median (IQR) time to dropout was 17 (9–26) weeks. The VIF was < 5.0 for all factors, therefore collinearity between factors was negligible. Within the set of investigated factors, women had a higher risk to dropout over the 6 months period compared to men (median time to dropout 15 vs 19 weeks, HR 1.58, 95% CI 1.06–2.36; Table 4). Low medication adherence (median time to dropout 16.5, IQR 8.5–26) compared to high (median time to dropout 18 weeks IQR 9–26), biological usage (15 weeks, IQR 8–26) compared to csDMARDs (18 weeks, IQR 9–26), a higher education level (16 weeks, IQR 8–26) compared to lower (18 weeks, IQR 7–26), and the patients with PsA diagnosis (16 weeks, IQR 8–26) compared to RA (20 weeks, IQR 10–26) had all a small but not statistically significant increased risk for dropout over 6 months. Since gender was significantly associated with time to dropout, we post-hoc stratified the analysis for men and women separately to assess if the set of factors associated with time to drop out differed between men and women. There were small but not significant differences found in hazard ratios for time to dropout for both men and women, see Table 1 and Figure 1 in the Additional file 1.

**Table 4:** Associations with time to dropout of reporting ePRO’s

Variable	Hazard ratio	95% CI		*p*
**Women**	**1.58**	**1.06**	**2.36**	**0.02**
Higher education	1.36	0.92	2.02	0.13
Biological usage^a^	1.18	0.83	1.68	0.35
High medication adherence	1.14	0.80	1.63	0.47
Diagnosis (relative to RA)				
PsA	1.14	0.77	1.69	0.53
AS	1.09	0.63	1.90	0.76
Smartphone usage	1.08	0.94	1.23	0.29
Charlson Comorbidity index	1.05	0.78	1.40	0.76
Resident in Amsterdam	1.02	0.72	1.44	0.93
Disease duration	1.01	0.99	1.02	0.49
patient-physician Interaction	1.00	0.92	1.09	1.00
Self-management	1.00	0.98	1.02	0.72
Age	0.99	0.96	1.02	0.39
RAPID3	0.97	0.89	1.06	0.51

Multivariable Cox-regression analysis where the hazard ratios are corrected for all factors in the model. N = 217 due to three patients missing baseline values for four factors. Sorted from highest to lowest hazard ratio

The bold values are statistically significant (*p* < 0.05)

*RA* rheumatoid arthritis, *PsA* psoriatic arthritis, *AS* ankylosing spondylitis, *RAPID3* routine assessment of patient index data 3 at baseline

^a^Compared to conventional Disease Modifying Antirheumatic Drugs

Patients who dropped out in the 1st month and 2nd to 3rd month reported significant lower mean SUS scores compared with adherent patients (respectively 67.6 for month 1 and 71.5 for month 2 to 3 vs 81.8 for adherent patients, *p* < 0.001). The SUS scores for patients dropped out in the 4th–6th month was lower, but this difference was not statistically different (78.2 *p* = 0.18).

### User activity ratio

**Fig. 3:**
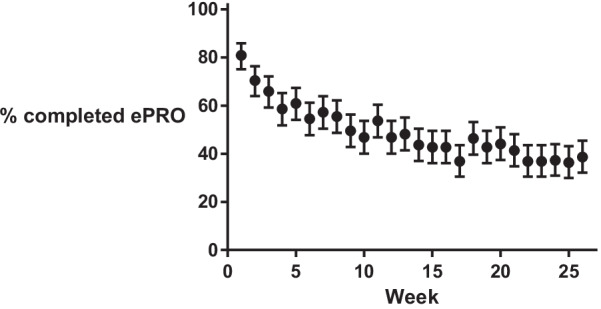
User activity ratio (% completed ePRO) with 95% confidence interval per week during the study

The UAR over 6 months was 49%. In the first week 81% completed the ePRO, which decreased to 39% in the last week. The decline was steepest in the first weeks of the study and consolidated from week 14, see Fig. 3.

Women had significant lower odds to complete ePRO’s compared to men (OR 0.66, 95%CI 0.43–1.00), see Table 5. Patients with a higher comorbidity index had higher odds to complete ePRO’s, but the result.

**Table 5:** Association of factors related with completing more ePRO’s

Variable	Odds ratio	95% CI		*p*
Comorbidity index	1.13	0.84	1.52	0.43
RAPID3	1.03	0.94	1.14	0.53
Self-management	1.01	0.99	1.03	0.40
Age	1.00	0.97	1.03	0.94
Residence in Amsterdam	1.00	0.69	1.45	0.99
Interaction patient-physician	0.99	0.91	1.08	0.79
Disease duration (years)	0.99	0.98	1.01	0.44
Smartphone score	0.91	0.79	1.05	0.20
High medication adherence	0.87	0.60	1.27	0.48
Diagnosis, compared to RA				
AS	0.84	0.46	1.54	0.58
PsA	0.82	0.53	1.27	0.38
Higher education level	0.77	0.51	1.16	0.21
Biological usage^a^	0.71	0.48	1.03	0.07
Women	0.66	0.43	1.00	**0.05**

Multivariable Generalized Estimated Equation model where the odds ratios are corrected for all factors in the model. The dependent variable is ePRO completed (yes/no), sorted from highest to lowest odds ratio. N = 217 due to three patients missing baseline values for four factors

The bold values are statistically significant (*p* < 0.05)

*RAPID3* routine assessment of patient index data 3 at baseline, *RA* rheumatoid arthritis, *PsA* psoriatic arthritis, *AS* ankylosing spondylitis

^a^Compared to conventional Disease Modifying Antirheumatic Drugs

was not statistically different. The lower odds to complete ePRO’s for biological usage, higher educational level, diagnosis, higher smartphone usage and higher medication adherence were all not statistically significant. Again, since gender was significant associated with the UAR, we post-hoc stratified for gender and repeated the analysis to assess if the factors are different associated to adherence for men and women. In the combination of factors, there were small but not significant differences in odds ratio to report ePRO’s for both men and women, see Table 2 in the Additional file 1.

## Discussion

This study investigated the association of a combined set patient- and clinical related factors with (non-)adherence to telemonitoring disease activity with a weekly ePRO in patients with inflammatory arthritis. Here we showed that of the 13 investigated factors, only gender was significantly associated with adherence: women had both higher chances to dropout over 6 months and lower odds to report the weekly ePRO’s.

The association between gender and adherence has been described in the widely accepted UTAUT model [12]. This model states that there are differences between genders in the mechanism of how they adopt and use new technologies such as apps for telemonitoring. Thus, a difference in usage in new technology between genders might be present if they are unintentionally easier to adopt for either men or women. The UTAUT describes that one of the differences is that women tend to rely more on supporting factors than men. Similar studies identified in a recent systematic review, approached patients actively when they stopped reporting ePRO’s to ask if they needed support, and did not find a significant association between adherence and gender [14]. In our study, the initiative to seek (technical) support for the app was given to the patients themselves, which may have contributed to the discrepancy in adherence between men and women.

Other factors could also account for the conflicting results regarding gender differences between our study and the results found in the systematic review [14]. For example, it is notable that Colls et al., and Jamilloux et al. included predominantly women (81% and 79%) [15, 30], Guzman et al. predominantly men (97%) [31], while Rosen et al. had a small sample size of 50 participants consisting of predominantly women (71%) [32]. Therefore, the included studies in the review may not have the proper men/women ratio for their sample size to establish a significant difference in adherence between men and women, which is different compared to our study, with a more balanced ratio between men and women.

If gender differences exist in adherence to traditional face-to-face follow-up visits is unknown, as we could not find any literature regarding this. However, we did find that the relationship between adherence and gender is frequently described considering medication administration, although the results are contradicting. While a systematic review towards adherence to biological treatment in patients with inflammatory arthritis (RA, PsA, AS) found that women were in general less adherent than men, a recent performed prospective cohort in patients with RA showed no significant difference between men and women (OR 0.90, 95CI 0.44–1.85) [33, 34]. Reasons why gender differences may be present in adherence to medication usage were not investigated.

This study was due to the quantitative nature also unable to identify reasons for why women had higher risks to dropout then men. However, we do hypothesize that by increasing the support for a telemonitoring program, we might be able to decrease the observed adherence gap between men and women in our population. Future prospective studies are necessary to corroborate our results and identify why women dropped out sooner. Furthermore, since our results suggests that gender differences may also exist in eHealth in rheumatic care, we advise that research groups need to investigate potential gender differences in adherence when developing new eHealth interventions such as telemonitoring disease activity.

Potentially vulnerable patients, such as patients with a lower level of self-management, older age, or lower education level, did not have an increased risk to dropout over the 6 months in our study. In contrary, patients with a higher education had a small (although not significant) higher risk to dropout. We found this to be remarkable. It could be that we could not identify these findings as the population of our study consisted for 50% of higher educated patients, and the patients reported a higher self-management (EC-17) baseline score compared with other patients found in literature [23]. Another reason may be that the education level does not play an important role in filling out ePRO’s [15], or it may be the result of selection bias as only 220 out of 825 (27%) invited patients participated in the study. An unintended selection bias is frequently observed in eHealth studies in rheumatology. For example, Colls et al. reported a remarkable high education level for their participants: > 80% attained college or an even higher educational level [15]. And Müskens et al. showed that RA patients participating in an eHealth platform tended to be younger and higher educated than patients who did not [35], which seems in line with our study population. Thus, although adherence was not lower for potential vulnerable patients in our study, their underrepresentation in eHealth studies suggests that it is urgent that future research focusses on how to make eHealth, and telemonitoring specifically, more accessible for all patients.

We found that patients who dropped out in the first 3 months reported a significant lower perceived usability of the app than adherent patients. A recent study showed that for optimal adoption of apps in rheumatic care, apps should be adjusted to the needs of rheumatic patients and their level of eHealth literacy [36]. A patient centered design is therefore deemed crucial [37]. Although the app used in this study was from the start designed with patients’ input, the system usability scores indicate that there are still patients who are unsatisfied with the usability of the app (mean SUS 67.6 for participants that dropped out early, compared to 81.8 for adherent participants), which may have influenced the adherence. Therefore, optimization of the app should be continued even after implementation with continuous feedback of patients to increase the usability, adoption, and adherence. We will perform focus group discussions in our study population to investigate how we can improve the app and overcome perceived barriers in the usage of the app.

There are other limitations to our study which should be noted. Firstly, with our study design we could not determine causality between the investigated factors and (non-)adherence. It is therefore unclear if an improvement in reported usability scores will lead to higher adherence. Secondly, the generalizability of studies investigating adherence to telemonitoring prospectively, and therefore also this study, is limited. This since adherence to telemonitoring with ePRO’s is assumed to be subject to a multitude of factors that are all in relation to each other, and which are unlikely to be the same between studies. For example, the used tool to collect ePRO’s is deemed to influence the amount of reported ePRO’s significantly, however every research group develops their own tool (app) to connect with their electronic medical records [38]. Therefore, our results may be different in other settings, even with an optimal internal validity. We countered the influence of the inter-factor associations as much as possible by analyzing the different factors as a group-set and incorporate as much relevant factors as we were able to. Still, the limited external validity should be considered if the results are extrapolated to other settings.

## Conclusion

Over 60% of the patients who telemonitored their disease activity in-between visits with a weekly ePRO over a 26-week period dropped out. Especially women stopped reporting the weekly ePRO’s sooner than men and had lower odds to report the weekly ePRO’s. Reasons why patients become non-adherent, as well as reasons to adhere to telemonitoring need to be investigated to improve the adoption of telemonitoring with ePRO’s in general, and for women specifically.

## Supplementary Information

**Additional file 1: Figure 1.** Proportion of participants who stop reporting ePRO’s over time, split for gender. **Table 1.** Hazard ratios for dropout, stratified for gender. **Table 2.** Odds ratio to complete an electronic patient reported outcomes, stratified for gender.

## Data Availability

The datasets used and/or analysed during the current study are available from the corresponding author on reasonable request.
