# Supplementary material for: Who stop telemonitoring disease activity and who adhere: a prospective cohort study of patients with inflammatory arthritis
Source: BMC Rheumatol. 2022 Nov 30;6:73. doi: 10.1186/s41927-022-00303-w (PMC9708135; doi:10.1186/s41927-022-00303-w)
Supplement: Supplementary file 1 — Additional file 1: Figure 1. Proportion of participants who stop reporting ePRO’s over time, split for gender. Table 1. Hazard ratios for dropout, stratified for gender. Table 2. Odds ratio to complete an electronic patient reported outcomes, stratified for gender. [file 41927_2022_303_MOESM1_ESM.docx]

## Supplementary material

Figure 1. Proportion of participants who stop reporting ePRO’s over time, split for gender.

| **Number at risk** |  |  |  |  |  |  |
| --- | --- | --- | --- | --- | --- | --- |
| **Women** | 121 | 121 | 82 | 65 | 50 | 39 |
| **Men** | 99 | 98 | 69 | 60 | 51 | 45 |

| Table 1. Hazard ratios for dropout, stratified for gender. | | | | | |  |  | |  | |  |
| --- | --- | --- | --- | --- | --- | --- | --- | --- | --- | --- | --- |
|  | Male | | | | Female | | | | | | |
| Variable | HR | 95%CI | | p | HR | 95%CI | | | | p | |
| Higher education | 1,26 | 0,63; | 2,51 | 0,52 | 1,43 | 0,85; | | 2,42 | | 0,18 | |
| Biological usage^1^ | 0,97 | 0,52; | 1,80 | 0,93 | 1,43 | 0,90; | | 2,27 | | 0,13 | |
| High medication adherence | 1,08 | 0,60; | 1,97 | 0,79 | 1,15 | 0,71; | | 1,85 | | 0,57 | |
| Diagnosis (relative to RA^2^) |  |  |  |  |  |  | |  | |  | |
| PsA^3^ | 1,53 | 0,73; | 3,20 | 0,26 | 0,97 | 0,58; | | 1,62 | | 0,91 | |
| AS^4^ | 1,60 | 0,58; | 4,37 | 0,36 | 1,04 | 0,50; | | 2,20 | | 0,93 | |
| Smartphone usage | 0,94 | 0,76; | 1,17 | 0,59 | 1,22 | 0,98; | | 1,46 | | 0,08 | |
| Charlson Comorbidity index | 0,93 | 0,60; | 1,45 | 0,76 | 1,25 | 0,78; | | 2,00 | | 0,36 | |
| Resident in Amsterdam | 1,10 | 0,61; | 1,97 | 0,76 | 0,98 | 0,57; | | 1,43 | | 0,65 | |
| Disease duration | 1,01 | 0,98; | 1,04 | 0,64 | 1,00 | 0,98; | | 1,03 | | 0,92 | |
| patient-physician Interaction | 0,96 | 0,83; | 1,10 | 0,55 | 1,01 | 0,89; | | 1,15 | | 0,54 | |
| Self-management | 1,00 | 0,97; | 1,04 | 0,86 | 0,99 | 0,96; | | 1,01 | | 0,32 | |
| Age | 0,99 | 0,94; | 1,04 | 0,72 | 0,99 | 0,95; | | 1,03 | | 0,51 | |
| RAPID3^5^ | 0,90 | 0,77; | 1,06 | 0,22 | 0,98 | 0,87; | | 1,11 | | 0,76 | |
| Multivariable Cox-regression analysis where the hazard ratios are corrected for all factors in the model. ^1^Compared to conventional Disease Modifying Antirheumatic Drugs; ^2^RA= rheumatoid arthritis; ^3^PsA= psoriatic arthritis; ^4^AS= ankylosing spondylitis; ^5^RAPID3= routine assessment of patient index data 3 at baseline. | | | | | | | | | | |  |

| Table 2. Odds ratio to complete an electronic patient reported outcomes, stratified for gender. | | | | | | | | |
| --- | --- | --- | --- | --- | --- | --- | --- | --- |
|  | Male | | | | Female | | | |
| Variable | OR | 95% CI | | p | OR | 95% CI | | p |
| Comorbidity index | 1,29 | 0,88; | 1,88 | 0,19 | 0,81 | 0,50; | 1,32 | 0,40 |
| RAPID3^1^ | 1,08 | 0,94; | 1,24 | 0,27 | 1,03 | 0,90; | 1,19 | 0,68 |
| Self-management | 0,99 | 0,95; | 1,04 | 0,77 | 0,99 | 0,95; | 1,04 | 0,77 |
| Age | 1,00 | 0,95; | 1,05 | 0,95 | 1,01 | 0,97; | 1,05 | 0,71 |
| Residence in Amsterdam | 0,84 | 0,46; | 1,52 | 0,56 | 1,22 | 0,73; | 2,06 | 0,45 |
| Interaction patient-physician | 1,07 | 0,93; | 1,23 | 0,32 | 0,92 | 0,84; | 1,03 | 0,15 |
| Disease duration (years) | 1,00 | 0,97; | 1,03 | 0,87 | 0,99 | 0,96; | 1,02 | 0,40 |
| Smartphone score | 1,03 | 0,85; | 1,25 | 0,75 | 1,02 | 0,81; | 1,28 | 0,69 |
| High medication adherence | 0,88 | 0,51; | 1,55 | 0,67 | 0,87 | 0,52; | 1,47 | 0,61 |
| Diagnosis, compared to RA^2^ |  |  |  |  |  |  |  |  |
| AS^3^ | 0,71 | 0,27; | 1,87 | 0,48 | 0,78 | 0,32; | 1,92 | 0,59 |
| PsA^4^ | 0,74 | 0,37; | 1,51 | 0,41 | 0,89 | 0,50; | 1,57 | 0,68 |
| Higher education level | 0,85 | 0,47; | 1,54 | 0,59 | 0,68 | 0,38; | 1,20 | 0,18 |
| Biological usage^5^ | 0,78 | 0,45; | 1,36 | 0,38 | 1,03 | 0,90; | 1,19 | 0,68 |
| Multivariable Generalized Estimated Equation model where the odds ratios are corrected for all factors in the model. The dependent variable is ePRO completed (yes/no). ^1^RAPID3= routine assessment of patient index data 3 at baseline; ^2^RA= rheumatoid arthritis; ^3^PsA= psoriatic arthritis; ^4^AS= ankylosing spondylitis; ^5^Compared to conventional Disease Modifying Antirheumatic Drugs. | | | | | | | | |
